# Xylazine-induced reduction of tissue sensitivity to insulin leads to acute hyperglycemia in diabetic and normoglycemic monkeys

**DOI:** 10.1186/1471-2253-13-33

**Published:** 2013-10-20

**Authors:** Yong-Fu Xiao, Bingdi Wang, Xiaoli Wang, Fenglai Du, Michael Benzinou, Yi-Xin Jim Wang

**Affiliations:** 1 Cardiovascular and Metabolic Disease Research, Crown Bioscience Inc, Science and Technology Park, 6 Beijing West Road, Taicang Economic Development Area, Jiangsu Province 215400, The People’s Republic of China

**Keywords:** Xylazine, Hyperglycemia, Monkey, Insulin, Glucose

## Abstract

**Background:**

The α_2_-adrenoceptor agonist xylazine as an anesthetic has been widely used either alone or in combination with other anesthetics, such as ketamine, in veterinary clinic and research. In the last decade xylazine has been used in drug abusers in certain geographic area. This study investigated the effects of xylazine on blood glucose level and insulin secretion in normoglycemic and insulin-dependent diabetic monkeys.

**Methods:**

Both adult cynomolgus (n = 10) and rhesus (n = 8) monkeys with either sex were used in the study. Xylazine (1–2 mg/kg) was administrated intramuscularly. Blood glucose, insulin, glucagon and glucagon-like peptide 1 in overnight-fasted monkeys were measured immediately before and after xylazine administration. The hyperinsulinemic-euglycemic clamp method was used in the study for assessing the potential mechanism of xylazine-induced hyperglycemia.

**Results:**

Xylazine administration increased the blood glucose levels from 58 ± 3 to 108 ± 12 mg/dL in normoglycemic (n = 5, *p <* 0.01) and from 158 ± 9 to 221 ± 13 mg/dL in insulin-dependent diabetic (n = 5, *p* < 0.01) monkeys and was not accompanied by any significant changes in blood insulin, glucagon, and glucagon-like peptide-1. Xylazine-induced hyperglycemia occurred within 10 min and reached the peak at 35 min after injection. Xylazine-induced hyperglycemia declined slowly in diabetic animals. The α_2_-adrenoceptor antagonist yohimbine was administrated to bring down the elevated glucose level to the pre-xylazine one in 4 out of 5 diabetic animals. To assess the potential mechanism, the hyperinsulinemic-euglycemic clamp was used to maintain a nearly saturated and constant insulin level for minimizing endogenous insulin glucoregulation. Xylazine administration decreased glucose infusion rate, from 14.3 ± 1.4 to 8.3 ± 0.8 mg/min/kg (n = 6, *p* < 0.01) in normoglycemic rhesus monkeys, which indicates that the glucose metabolic rate (M rate) was decreased by xylazine. In addition, after clamping blood glucose level in a range of 55 to 75 mg/dL for 40 min with constant glucose infusion, xylazine administration still increased blood glucose concentration.

**Conclusions:**

We conclude that xylazine administration induces hyperglycemia in normoglycemic and insulin-dependent diabetic monkeys potentially via stimulation of α_2_-adrenoceptors and then reducing tissue sensitivity to insulin and glucose uptake.

## Background

Xylazine is an α_2_-adrenoceptor agonist and shares the similar pharmacological properties with clonidine [1-4]. It has been widely used as a drug for sedation and analgesia in veterinary clinic and non-human mammal research for several decades [5,6]. It can relieve pain and relax skeletal muscle. Xylazine is often co-administrated with ketamine to provide reliable anesthesia effects [7,8]. The sedative and muscle-relaxing properties of xylazine can be beneficial in reducing ketamine-induced side effects, such as tremor and muscle rigidity. It also can reduce animal gastric and intestinal motility during gastrointestinal surgery or endoscopy. Its analgesic action is less than 30 min, but its sedative effect can last 2 hrs. Xylazine has adverse effects (e.g., cardiac conduction disturbances, bradycardia, and myocardial depression) similar to other α_2_ agonists. These adverse effects can be attenuated, blocked or reversed by adrenergic α_2_-receptor antagonists such as yohimbine [9,10].

Maintaining blood glucose homeostasis involves complex neurohumoral regulation. Stress can increase blood glucose by changing several hormones including insulin, glucagon, GLP-1, and catecholamines [11,12]. Administration of the potent α_2_ agonist xylazine can result in a neurohumoral imbalance which affects blood glucose. In fact, several studies demonstrate that administration of xylazine increases blood glucose in various animal species, including dogs, cats, rats, and mice [12-16].

Currently, xylazine is not authorized for human use. However, early studies showed that xylazine induced sedation, muscle relaxation, and analgesia in healthy volunteers [17,18]. All volunteers in those studies exhibited a significant reduction of blood pressure and heart rate. Interestingly, xylazine effectively lowered blood pressure and heart rate in some hypertensive patients [18,19]. This veterinary anesthetic compound has been used as a new recreational drug among drug abusers in certain geographic areas [20-24]. Chronic use of this substance induces physical dependence and open skin ulcers or abscesses [25]. More severe intoxication has been reported in xylazine users [19] and post-mortem examinations have attributed the death to xylazine (26–29). Medical examination in some drug-related deaths detected xylazine concurrently [26-29].

Previous studies show that xylazine increases blood glucose in both small and large animals [12-14]. However, the effects of this drug on blood glucose homeostasis in non-human primates (NHPs) are unclear. The present study investigates the effects of xylazine on blood glucose in fasted, ketamine-anesthetized monkeys with or without diabetes. We also assessed xylazine’s effects on the secretion of insulin, glucagon, and glucagon-like peptide 1 (GLP-1). Understanding xylazine pharmacology and adverse effects in NHPs can provide useful information regarding its use in veterinary clinics and animal research as well as for proper therapy of abusers intoxicated with this α_2_-adrenoceptor agonist.

## Methods

### Animal care and procedures

Experiments were carried out in cynomolgus (Table 1) and rhesus monkeys of either sex. These monkeys were individually housed and maintained in our animal facility in accordance with guidelines approved by the Association for Assessment and Accreditation of Laboratory Animal Care (AAALAC). Animals had continuous access to water *ad libitum* and controlled access to food. Room temperature was maintained at ∼ 21°C. The animals were maintained on a 12 hr light/dark cycle with lights off from 6 PM to 6 AM. The monkeys were maintained with a complete nutritionally balanced diet (Shanghai Shilin Biotechnology, Inc., Shanghai, China) and enriched with seasonal fruits and vegetables. The experimental protocol was approved by the Institutional Animal Care and Use Committee (IACUC) of Crown Bioscience, Inc.

**Table 1:** Characteristics of the diabetic and normoglycemic cynomolgus monkeys used

**Monkey**	**n**	**Age (year)**	**Body weight (kg)**	**Fasting glucose (mg/dL)**	**Fasting insulin (μIU/ml)**
Normal	5	6.8 ± 0.3	6.7 ± 0.7	47.9 ± 5.4	23.9 ± 3.9
Diabetic	5	18.3 ± 0.8***	6.0 ± 0.7	183.6 ± 29.8***	12.1 ± 0.7***

Values are expressed as the mean ± SEM. Normal, normoglycemic monkeys; Diabetic, insulin-dependent diabetic monkeys; ***, *p* < 0.001; vs. normal.

On the experimental day each monkey was fasted overnight and received ketamine (10 mg/kg, Fujian Gutian Pharmaceutical Co. Ltd., Fujian, China) intramuscular administration. Sedation was maintained with additional ketamine (5 mg/kg) injection as needed. Xylazine and yohimbine (Sigma-Aldrich Co., St. Louis, MO, USA) were given intramuscularly for testing their effects on blood glucose. Body temperature was maintained during each experiment at ~37°C by a thermostatically controlled warm water–circulating pad placed beneath the body. Food and water were provided again after experimental animals were returned to their cages and fully recovered from anesthesia.

### Blood collection and handling

Whole blood samples (1–2 ml/per time) at various time points after anesthesia or compound treatment were collected from a plastic needle inserted into an arm vein. Samples were collected into an EDTA-washed (0.5 M EDTA, pH 8.0, Gibco, Invitrogen Corporation, Grand Island, NY, USA) 2.5-mL disposable syringe and transferred immediately into a 5-ml Monoject™ blood collection tube containing 7.5 mg EDTA (Sherwood Medical, St. Louis, MO, USA). The collection tube also contained aprotinin (Sigma-Aldrich Co., St. Louis, MO, USA) for reduction of protein degradation and DPP IV inhibitor (EMD Millipore Corporation, Billerica, MA, USA) for prevention of GLP-1 degradation. Blood samples were centrifuged within 30 min at 4°C, 3000 *g* for 10 min and then the plasma was separated following standard protocols established in our laboratory. The plasma samples were kept in a freezer at -80°C prior to analysis of glucose, insulin, glucagon, and GLP-1 concentrations.

### Intravenous glucose tolerance test (ivGTT)

To evaluate the β-cell function ivGTT was performed in the diabetic and normoglycemic monkeys according to the method reported previously [30,31]. The animals were fasted for 16 hrs and anesthetized with an initial dose of ketamine at 15 mg/kg (i.m.) with additional doses during the procedure if needed. The cephalic and/or saphenous veins were cannulated separately for glucose infusion and blood collection. Glucose (0.25 g/kg = 0.5 ml/kg of 50% dextrose) was intravenously infused during 30 sec and the system was flushed with 5 ml heparinized saline to remove residual glucose. Blood was collected immediately before and at 3, 5, 7, 10, 15, 20, 30 min after glucose infusion. Blood samples were immediately transferred to heparinized and chilled tubes on ice. Plasma was then separated and stored at -80°C for subsequent assays.

### Hyperinsulinemic-euglycemic clamp

Hyperinsulinemic-euglycemic clamp analysis was performed in 16-hr-fasted monkeys under ketamine-anesthesia. Cannulation of the cephalic and/or saphenous veins was conducted for insulin and glucose infusion, and blood drawing for glucose measurement. Insulin (biosynthetic human insulin, Novo Nordisk, Denmark) was diluted to 300 mU/ml by isotonic saline to which 2 ml of the subject’s blood per 50 ml was added in order to avoid adhesion of insulin to the syringe plastic surface. Insulin infusion at various rates was given during the 1st 10 min to quickly adjust blood glucose near a targeted level. The infusion rate for the hyperinsulinemic-euglycemic clamp was then maintained at 40 mU/m^2^ Surface Area*min as reported previously [32].

A variable amount of 20% D-glucose was intravenously infused to maintain blood glucose. Blood samples were taken every 5 min and glucose was measured by a glucose analyzer (Accu-Chek Active, Roche Diagnostics, Indianapolis, IN, USA) to allow adjustment of the glucose infusion rate accordingly. In the 1st set of experiments, when blood glucose was adjusted and balanced for approximately 120 to 150 min and then for a 40-min stable period of glucose levels clamped in a range of 55 to 75 mg/dL under constant infusion of glucose, xylazine was injected intramuscularly. Blood glucose concentration was monitored for another 30 to 40 min after xylazine injection. In the 2nd set of experiments, xylazine was injected after blood glucose level was adjusted for 95 to 135 min and then maintained in the range of 55 to 75 mg/dL for 40 min. The glucose infusion rate was adjusted after xylazine treatment to clamp blood glucose in the range of 55–75 mg/dL. The difference of the glucose metabolic rates (M rate) was calculated from the glucose infusion rates before and after xylazine treatment.

### Data analysis

Data were expressed as mean ± SEM. Statistical significance for multiple observation parameters in the same group was determined by One-way Analysis of Variance (ANOVA). If statistical significance of differences was detected, then Tukey’s Multiple Comparison Test (GraphPad Software, Inc., La Jolla, CA, USA) was also conducted. The comparison between diabetic and normoglycemic groups was tested by the un-paired *t*-test. Statistical significance was considered if *p* value was <0.05.

## Results

### Selection of diabetic monkeys

As the effects of xylazine on blood glucose had not been studied in NHPs previously, we selected diabetic and normoglycemic monkeys for this study. Based on blood glucose (<90 mg/dL) and other biochemistry tests, five healthy young cynomolgus monkeys were selected for the normoglycemic group (Table 1). The other five cynomolgus monkeys were selected from spontaneously naturally developed and already insulin-treated diabetic animals with blood glucose >150 mg/dL. Compared with controls, the diabetic animals were significantly older (Table 1). They received insulin treatment daily because of their high blood glucose, low insulin, and losing body weight if not insulin-treated.

**Figure 1:**
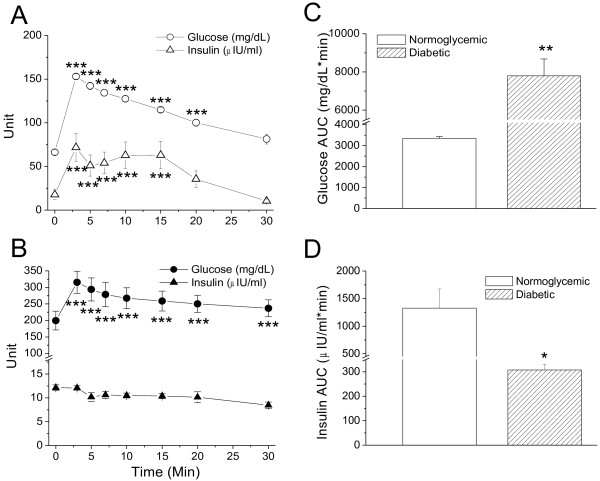
**Intravenous glucose tolerance test (ivGTT) in normoglycemic and insulin-dependent diabetic cynomolgus monkeys (Table**1**).** Plasma glucose and insulin levels were measured at the scheduled time points in normoglycemic (panel ***A***, n = 5) and insulin-dependent diabetic (panel ***B***, n = 5) monkeys. Panel ***C*** shows the glucose AUCs of the ivGTTs for the nomorglycemic (blank bar) and diabetic (strip bar) monkeys. Panel ***D*** shows the insulin AUCs of the ivGTTs for the normoglycemic (blank bar) and diabetic (strip bar) monkeys. *, *p* < 0.05; **, *p* < 0.01, ***, *p* < 0.001; vs. the level at time zero or vs. the normoglycemic group.

The selected animals were characterized by intravenous glucose tolerance test (ivGTT) and insulin secretion assay. Figure 1A shows that blood glucose and insulin were significantly increased immediately after glucose challenge in normoglycemic monkeys who had normal β-cell function. The significant increase in blood glucose lasted 20 min and 15 min in insulin and then returned to the control levels within 30 min. However, in the insulin-dependent diabetic monkeys only blood glucose was significantly increased without significant alteration of insulin (Figure 1B). The glucose AUC (area under the curve) was significantly higher in the diabetic monkeys than in the normoglycemic ones (Figure 1C, *p* < 0.01). In contrast, the insulin AUC was significantly lower in the diabetic monkeys than in the normoglycemic ones (Figure 1D, *p* < 0.05). These results indicate that pancreatic function was normal in the normoglycemic monkeys and was significantly impaired in the diabetic ones. To look at the differences between the strains and their xylazine responses, eight normal rhesus monkeys were also used for xylazine test during glucose clamp.

### Xylazine-induced hyperglycemia in diabetic and normoglycemic monkeys

**Figure 2:**
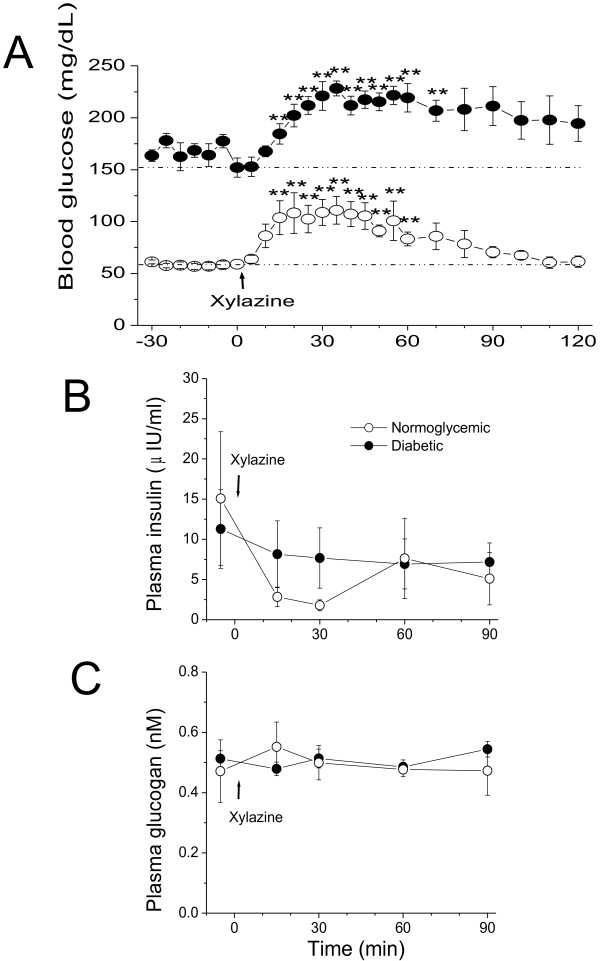
**Hyperglycemic effects of xylazine in fasted, ketamine-anesthetized cynomolgus monkeys.** Panel ***A*** shows the time-dependent effects of xylazine in normoglycemic (open circle, n = 5) and insulin-dependent diabetic (solid circle, n = 5) monkeys. The dotted lines show the pre-xylazine glucose levels in normoglycemic and insulin-dependent diabetic monkeys. The time-dependent effects of xylazine on plasma insulin and glucagon are shown in panel ***B*** and ***C***, respectively. **↑**, the time point that xylazine (1–2 mg/kg) was injected intramuscularly. **, *p* < 0.01; vs. the baseline level at time zero.

After verification of the pancreatic functions in the selected diabetic and normoglycemic monkeys, the effects of xylazine on blood glucose were investigated. Blood glucose was measured immediately before and at various time points over the 2 hr observation (Figure 2A). Intramuscular injection of xylazine (1–2 mg/kg) significantly increased blood glucose in a time-dependent manner in the normoglycemic monkeys (Figure 2A, open circle). An initial rise of blood glucose was observed at 5 to 10 min after xylazine injection. The increase reached the peak of 108 ± 12 mg/dL from the baseline of 58 ± 3 mg/dL (*p* < 0.01) at approximately 35 min following xylazine administration. Hyperglycemia began to decline approximately 45 min after xylazine injection. Blood glucose in all of the normoglycemic monkeys gradually returned to the pre-xylazine levels within 2 hrs (Figure 2A, open cycle).

Xylazine-induced hyperglycemia was also observed in the diabetic monkeys (n = 5). Xylazine administration (1–2 mg/kg) increased blood glucose from the baseline of 158 ± 9 mg/dL to 221 ± 13 mg/dL at approximately 35 min following the injection (*p* < 0.05, Figure 2A, filled cycle). The initial rise and time to the peak after xylazine administration were very similar to those observed in the normoglycemic monkeys. However, the initial decline of blood glucose was delayed to approximately 60 min after xylazine administration. The decline toward the pre-xylazine level was much slower in the diabetic monkeys than in the normoglycemic ones (Figure 2A, dot line). Blood glucose remained high 2 hrs after xylazine administration (Figure 2A, filled circle). Only one out of 5 diabetic monkeys gradually returned to the pre-xylazine glucose level at 2 hr after xylazine administration. The other diabetic animals retained their elevated glucose levels 2 hrs after xylazine injection and the α_2_-adrenoceptor antagonist yohimbine (1–4 mg/kg) was injected to restore blood glucose toward the pre-xylazine levels for safety reason.

### Effects of xylazine on glucoregulatory hormones

To assess the possible mechanism of xylazine-induced hyperglycemia, plasma insulin, glucagon, and GLP-1 were measured in both diabetic and normoglycemic monkeys. As shown in Figure 2B (open cycles), the baseline level of plasma insulin in the normoglycemic monkeys was 15.1 ± 8.3 μIU/ml (n = 3). Xylazine administration (1–2 mg/kg) did not cause statistically significant alterations of plasma insulin in the non-diabetic monkeys (*p* > 0.05). Compared to the non-diabetes, the plasma insulin level was relatively lower in the diabetic monkeys (11.2 ± 4.8 μIU/ml, n = 5) and remained unchanged during 90-min observation after xylazine administration (Figure 2B, solid cycle).

The mean plasma glucagon levels were 0.47 ± 0.10 nM (n = 3) for the normoglycemic and 0.51 ± 0.02 nM (n = 5) for the insulin-dependent diabetic monkeys. Xylazine administration (1–2 mg/kg) did not alter the levels of plasma glucagon in either group (Figure 2C, *p* > 0.05). In addition, xylazine had no statistically significant effects on plasma GLP-1 level in both diabetic and normoglycemic monkeys. The baseline plasma GLP-1 level was 1.32 ± 0.14 pM (n = 3) for the normoglycemic monkeys and 2.52 ± 0.95 pM (n = 5) for the diabetic ones. The plasma GLP-1 level was 1.50 ± 0.64 pM for the normoglycemic monkeys and 2.43 ± 1.39 pM for the diabetic animals at 30 min after xylazine injection.

### Effects of xylazine on blood glucose during hyperinsulinemic-euglycemic clamp

**Figure 3:**
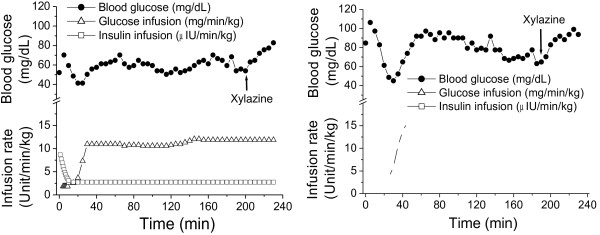
**Effects of xylazine on blood glucose concentrations in two fasted normoglycemic rhesus monkeys during the hyperinsulinemic-euglycemic clamp.** Insulin was given with an initial bolus infusion to reduce blood glucose close to a targeted level followed by a constant infusion rate of 40 mU/m^2^ Surface Area*min (open squares) for maintaining hyperinsulinemia. Glucose was infused simultaneously with adjustable rates to clamp blood glucose in the euglycemic range of 55 – 75 mg/dL (open triangles). Xylazine (2 mg/kg) was injected intramuscularly after 40-min euglycemia stabilization to observe its effects on blood glucose when glucose was infused at a constant rate.

To exclude or minimize the influence of endogenous insulin on xylazine-induced hyperglycemia and also to see the effects of xylazine on blood glucose in another monkey strain (rhesus), the hyperinsulinemic-euglycemic clamp was performed in normoglycemic rhesus monkeys of either sex (n = 8). Their mean age was 10.7 ± 1.1 years with the body weight of 7.7 ± 0.8 kg. Compared with the normoglycemic cynomolgus monkeys (Table 1, 47.9 ± 5.4 mg/dL), their blood glucose was significantly higher (86 ± 4.6 mg/dL) probably due to their older ages. They were intravenously infused with a constant dose of insulin following an initial bolus infusion in the 1st 10 min (Figure 3, open square). Glucose was simultaneously infused with rates adjusted until blood glucose was maintained in a range of 55 to 75 mg/dL under a constant infusion rate (Figure 3, open triangle). Prior to xylazine injection, a 40 min period (from 150–190 min after the initiation of the clamp, Figure 3) of stable blood glucose ranging from 55 to 75 mg/dL was reached (Figure 3). Administration of xylazine (2 mg/kg) significantly increased blood glucose level, from 55 mg/dL (left panel) and 64 mg/dL (right panel) for pre-xylazine to the peak of 83 mg/dL (left panel) and 99 mg/dL (right panel), respectively, despite the glucose infusion rate being unchanged. These results indicate that xylazine still produced hyperglycemia in the presence of clamp-maintained hyperinsulinemia which maximally inhibited endogenous negligible insulin secretion.

**Figure 4:**
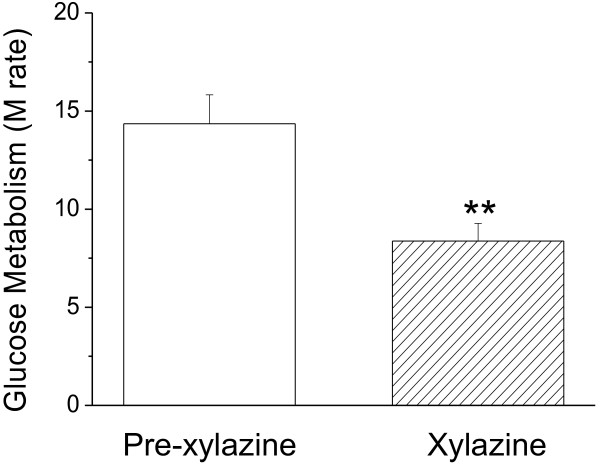
**Effects of xylazine on the glucose metabolic rate in fasted normoglycemic rhesus monkeys.** Hyperinsulinemia was maintained by insulin infusion at a constant rate of 40 mU/m^2^ Surface Area*min. Glucose was infused simultaneously to adjust and balance blood glucose for a period of 95 ± 11 min and then to achieve euglycemia (55 – 75 mg/dL) stably for 40 min with a constant infusion rate. Xylazine (2 mg/kg) was then injected intramuscularly. To hold blood glucose concentration constantly in the pre-xylazine range (55 – 75 mg/dL), the glucose infusion rate was decreased during next 40 min after xylazine administration. The glucose infusion rates were calculated as the glucose metabolic rates (M rates) at the times before (Pre-xylazine) and after (Xylazine) xylazine administration. **, *p* < 0.01; vs. Pre-xylazine.

To further investigate the role of insulin in xylazine-induced hyperglycemia, another set of experiments was conducted in normoglycemic monkeys who were clamped for maintaining hyperinsulinemia to minimize endogenous insulin glucoregulation. When blood glucose in these animals reached 40-min stabilization in a range of 55 to 75 mg/dL under constant glucose infusion, xylazine (2 mg/kg) was intramuscularly administrated. In order to hold the blood glucose level in the constant range of 55 to 75 mg/dL by the negative feedback principle, the glucose infusion rate was significantly reduced, from 14.3 ± 1.4 mg/min/kg for pre-xylazine (Figure 4, open bar) to 8.3 ± 0.8 mg/min/kg for post-xylazine (Figure 4, striped bar, n = 6, *p* < 0.01). Under these steady-state conditions of normoglycemia, the glucose infusion rate equals glucose uptake of the body and is therefore a measure of tissue sensitivity to exogenous insulin. These results support our hypothesis that xylazine-induced hyperglycemia most likely resulted from a reduction of tissue sensitivity to insulin, because blood insulin was stably maintained at a high level by a constant infusion.

## Discussion

The main finding of this study is that xylazine administration produced an acute hyperglycemia without significant changes in blood insulin, glucagon, and GLP-1 in both insulin-dependent diabetic and normoglycemic monkeys. As xylazine is widely used either alone or combined with other anesthetics, such as ketamine, in various animal research, its hyperglycemia can have an obvious impact on experimental results, especially in diabetic and metabolic research. Xylazine is commonly used as an anesthetic and analgesic in veterinary clinics [5,6]. Also, due to increasing amongst drug abusers [20-22], xylazine-induced hyperglycemia potentially becomes a clinically relevant issue, especially in diabetic subjects.

Xylazine-induced hyperglycemia was reported previously in various species, including dogs [11], cattle [33,34], and rats [12,35]. However, these studies were performed in normoglycemic animals. These studies led to the hypothesis that the inhibition of insulin secretion plays a critical role in the hyperglycemia. The present study attempts to test this hypothesis in a series of 3 experiments. First, we used naturally developed diabetic monkeys who required insulin treatment because their insulin secretory function was greatly diminished. Our data clearly demonstrate that xylazine not only produced profound hyperglycemia in the normoglycemic monkeys, but also in the diabetic animals (Figure 2). Secondly, blood insulin, glucagon, and GLP-1 exhibited no significant changes during the xylazine-induced hyperglycemic period (Figure 2). Thirdly, xylazine still caused hyperglycemia (Figure 3) and decreased glucose uptake (M rate, Figure 4) during hyperinsulinemic-euglycemic clamp. These results are consistent with those reported in foals that insulin was not significantly changed during xylazine-produced hyperglycemia [36]. However, xylazine-induced hyperglycemia in rats, sheep, cattle, and dogs is associated with a reduction of insulin secretion [35,37-41], while a significant rise in plasma insulin levels occurs in horses [42]. These inconsistent results may be due to different animal species used. Our results suggest that xylazine-induced hyperglycemia results from the decrease of tissue sensitivity to insulin, which leads to the reduction of tissue glucose uptake and utilization.

Xylazine is an analogue of the α_2_-adrenergic agonist clonidine. The effects of activation of α_2_-adrenoceptors on blood glucagon are inconsistent and whether the hyperglycemic effect of xylazine involves glucagon is not clear [37,43]. Previous studies in rats showed that xylazine significantly increased blood glucagon, which was not affected by the α_2_-adrenergic antagonist yohimbine [1,12,35]. The unchanged glucagon level found in our present study is consistent with the results previously reported in dogs [11]. Glucagon thus seems not so critical for xylazine-induced hyperglycemia at least in NHPs in this study. Furthermore, blood GLP-1 was also not altered after xylazine administration in the present study. Therefore, it is possible that the cause of xylazine-induced hyperglycemia results from stimulation of α_2_-adrenoceptors and then modifying other stress hormones, such as ACTH and GH, which were not measured in our study. However, xylazine has been reported to increase the release of ACTH and GH in cattle and dogs [3,39].

It is unclear whether xylazine itself could increase hepatic glucose production (glycogenolysis and gluconeogenesis) and then cause hyperglycemia. However, as xylazine-induced hyperglycemia observed in the present study was conducted in fasted monkeys which had reduced glycogen stores [44], the contribution of glycogenolysis to the hyperglycemia was very unlikely, especially in fasted insulin-dependent diabetic monkeys. In addition, the level of blood glucagon (the stimulating hormone of glycogenolysis and gluconeogenesis) was not increased in the presence of xylazine (Figure 2C). Therefore, xylazine-induced hyperglycemia was unlikely via an increase in hepatic glucose production.

Compared with the insulin-dependent diabetic monkeys, the decline of xylazine-induced hyperglycemia was faster and blood glucose returned to the pre-xylazine level within 90 min in normoglycemic monkeys (Figure 2A). In contrast, blood glucose remained elevated during the entire observation period of 120 min in the diabetic monkeys. The specific α_2_-adrenoceptor antagonist yohimbine had to be given in 4 out of 5 insulin dependent diabetic monkeys to decrease their blood glucose to the pre-xylazine level for animal safety reason. These results suggest that xylazine-induced hyperglycemia is mediated, at least partially, via stimulation of α_2_-adrenoceptors. Lattermann and colleagues reported that blood glucose concentrations were significantly increased in patients during and after lower abdominal surgery [45]. However, compared with control patients (saline), intraoperative glucose plasma concentrations were even higher in the patients who received clonidine (1 μg/kg) 30 min before induction of general anesthesia. The adverse effects of hyperglycemia can be reflected in animal models of myocardial infarction [46] and in patients after acute myocardial infarction [47], stroke [48], and cardiac surgery [49]. Therefore, great care should be taken to avoid using an agent which causes hyperglycemia and influences outcome.

Due to the difficulty of obtaining traditional illicit drugs, consumption in drug abusers is turning towards less restricted compounds. Xylazine, the veterinary sedative anesthetic, was confirmed as the anesthetic substance used in Puerto Rico by testing exchanged needles in 29 sites in 11 municipalities [22]. Xylazine used as adulterants in heroin was also found in drug related deaths in Philadelphia, Pennsylvania [20,50]. An 18-year-old man after inhaling xylazine showed chills and dizziness followed by sweating, gait instability, palpitations and syncope with bradycardia and hypotension. More cases of toxicity caused by xylazine consumption have been documented in humans, occasionally resulting in death [18,20,28]. Xylazine users could become apneic and require intubation and mechanical ventilation. Two critical issues about chronic use of xylazine are the physical dependence and the noticeable open skin ulcers [6]. These ulcers are referred to as abscesses and are a serious health concern. The pain caused by the ulcers promotes further injections of xylazine because of its sedative/anesthetic effects. These open skin ulcers emit a strong odor, ooze, and in severe cases limit the mobility of the extremities with a possibility of amputation [25]. In xylazine abusers (generally male with a mean age of 30 years) 35% have skin lesions, which leads to more social exclusion. It is unclear whether the skin ulcers result from the hyperglycemic effect of xylazine in abusers, because extremity infection in diabetic patients is common and severe, sometimes difficult to cure [22]. More experiments are thus required to elucidate how xylazine induces hyperglycemia at the cellular and molecular levels, which may have clinical relevance.

## Conclusions

In conclusion, the anesthetic xylazine can cause acute hyperglycemia with no significant alterations of blood insulin, glucagon, and GLP-1 in normoglycemic and insulin-dependent diabetic non-human primates. Such hyperglycemia is most likely via xylazine stimulation of α_2_-adrenoceptors and subsequent reduction of tissue sensitivity to insulin and glucose uptake.

## Competing interests

All of the authors are employees of Crown Bioscience, Inc.

## Authors’ contributions

YXW and MB designed the experiments, BW, XW and FD collected the data, XW and YFX analyzed the data, YFX, MB and YXW drafted the manuscript. All authors read and approved the final manuscript.

## Pre-publication history

The pre-publication history for this paper can be accessed here:

http://www.biomedcentral.com/1471-2253/13/33/prepub
